# Early Ontogeny of Cichlids Using Selected Species as Examples

**DOI:** 10.3390/ani14081238

**Published:** 2024-04-20

**Authors:** Radosław Piesiewicz, Jan Krzystolik, Agata Korzelecka-Orkisz, Adam Tański, Krzysztof Formicki

**Affiliations:** Department of Hydrobiology, Ichthyology and Biotechnology of Animal Reproduction, West Pomeranian University of Technology, 70-310 Szczecin, Poland; jaszko.krzystolik@gmail.com (J.K.); agata.korzelecka-orkisz@zut.edu.pl (A.K.-O.); adam.tanski@zut.edu.pl (A.T.); krzysztof.formicki@zut.edu.pl (K.F.)

**Keywords:** fish embryogenesis, ornamental fish, eggs characteristics, breeding behavior, alevins characteristics

## Abstract

**Simple Summary:**

The aim of this study was to provide a detailed characterization of the reproductive strategy, embryonic development, and larval development of three fish species from the genus Cichlasoma: the green terror (*Andinoacara rivulatus*), the red discus (*Symphysodon discus*), and the jaguar cichlid (*Parachromis managuensis*). Eggs for the study were obtained from five pairs of each species (300 eggs from each female) and incubated at 26 °C. Developing eggs were observed from fertilization to larval hatching, up to the complete absorption of the yolk sac. The results of the study will contribute to the development of reproductive biotechnology for the studied fish, enabling effective and easy breeding of these fish under controlled conditions. This will not only meet the demand for these species in the aquarium trade but also protect them in their natural habitat by discontinuing fishing activities.

**Abstract:**

The purpose of this study was to characterize in detail the reproductive strategy, course of embryogenesis, and development of larvae in three species of fishes of the genus *Cichlasoma*: the green terror (*Andinoacara rivulatus*), the red discus (*Symphysodon discus*), and the jaguar cichlid (*Parachromis managuensis*). Eggs for the study were obtained from five pairs of each species (300 eggs from each female) and incubated at 26 °C. The developing eggs were observed under a microscope (Carl Zeiss Stereo Discovery. V12 and Nikon 2000SE software (NIS-Elements F 4.30.01 64-bit) from fertilization to larval hatching until complete yolk-sac resorption. The largest average number of eggs per female was found in the jaguar cichlid ($\bar{x}$ = 2991 eggs), a smaller average number of eggs was shown in the green terror ($\bar{x}$ = 922 eggs), and the red discus showed the smallest average number of eggs ($\bar{x}$ = 300 eggs). There were significant differences in the sizes of the eggs of the studied species: jaguar cichlid eggs were the smallest (1.060 ± 0.05 mm^3^), red discus eggs were larger (1.070 ± 0.07 mm^3^), and green terror eggs were the largest (1.365 ± 0.16 mm^3^). The embryogenesis time in the red discus was 2132 °H (82 Hpf), in the green terror it was 2158 °H (83 Hpf), and the longest in the jaguar cichlid was 2470 °H (87 Hpf). At the end of embryogenesis, the average size of the larvae after hatching was measured (red discus $\bar{x}$ = 4.346 mm, green terror $\bar{x}$ = 5.203 mm, and jaguar cichlid $\bar{x}$ = 5.301 mm) and the time of yolk-sac resorption from the moment of hatching to the transition from endogenous to exogenous feeding was determined (jaguar cichlid 5 days, green terror 6 days, and red discus 3 days). The results of this study may contribute to the development of reproductive biotechnology for the studied fishes that could be used in aquaculture and, thus, help protect them in their natural habitats.

## 1. Introduction

Central and South America are home to many species of tropical fish, which, due to their rich body coloration and representative appearance, are a relatively common object of interest for aquarists. The cichlids of the genus *Cichlasoma* from these regions are of great interest not only for their rich coloration but also for their interesting reproductive behavior [1]. Three representatives of this genus, the jaguar cichlid (*Parachromis managuensis* Günther, 1867) from Central American waters, as well as the green terror (*Andinoacara rivulatus* Günther, 1860) and the red discus (*Symphysodon discus* Heckel, 1840) from South American waters, inhabit similar biotopes in the wild.

In Central America, the jaguar cichlid is found from the waters of the Ulua River (Honduras) to the Matina River (Costa Rica) [2,3]. In its natural habitat, it inhabits water bodies (lakes, rivers, and estuaries) with low flow and water temperatures of 25–30 °C [4,5,6,7,8]. The Jaguar cichlid shows great resilience to adverse environmental conditions, being able to survive in water temperatures up to 36 °C, or when oxygen in the environment drops to low levels [4], as well as being able to survive small increases in salinity [9,10]. This fish is found and reproduces in coastal waters on rocky substrates [5]. In its natural habitat, it is very aggressive and territorial—it will not tolerate either conspecifics or fish of other species in its vicinity. In the wild, its diet often includes invertebrates, although it usually comprises small fish [6], which has led to the species being used to limit and control the size of populations of other fish, especially invasive fish such as tilapia [11]. Jaguar cichlids grow to 65 cm in the wild [12]. The differences between males and females are clearly marked—males are slightly larger and their dorsal fin is triangular with a sharp tip, while females have a dorsal fin in the upper part of an oval shape. A characteristic feature of this species is the age-dependent change in coloration. Young, immature individuals have a silver coloration with distinct black vertical stripes, the first of which is located just behind the mouth; these stripes disappear on the ventral side. Almost all fins, except the pectoral fins, are intensely black. As they grow, the body coloration changes quite dramatically. The vertical black stripes disappear, leaving only some spots on the sides of the body along the lateral line. The fins change from black to a pattern of tiny black stripes on an olive-green background. The pattern on the body resembles the coloration of a jaguar, hence the English name for this fish [9].

The green terror occurs naturally in South American waters in Ecuador and Peru [13,14,15,16]. It is found in shallow, warm, slow-moving rivers where it inhabits coastal zones [17] These fish require a hard substrate for reproduction, consisting mainly of large stones. During spawning, they show great aggression towards conspecifics and other fish [18]. The green terror grows up to 20 cm [14]. Males of this species are significantly larger and more intensely colored than females, and their characteristic feature is a large fatty forehead hump. Under natural conditions, this hump is only visible during spawning but in aquariums, it is always visible due to the frequent spawning of the males. This species has an exceptionally beautiful coloration—rows of iridescent purple spots run evenly along the green–blue sides. The center of the trunk is marked by a large black spot on each side. The elongated dorsal fin and paddle-shaped caudal fin are bordered by broad, bright orange stripes. On the snout, narrow zigzag blue stripes extend from the mouth. The male is also distinguished from the female by the presence of a single distinctly elongated and intensely colored ray on the dorsal fin.

In the wild, the red discus occurs in aquatic areas of Brazil, where it mainly inhabits the Amazon River and its tributaries [19]. It is often found in the same habitats as the closely related *Symphysodon aequifasciatus*. During the dry season, the red discus forms large schools of hundreds of individuals. It inhabits coastal areas of the water, choosing sites with fallen tree branches and twigs [20]. During the rainy season, when large areas of land are flooded, these fish migrate to these areas where they find abundant food and breeding sites with suitable substrate [21]. The spawners spawn on trees that have been felled in the water. Both parents care for the spawned eggs and later the fry. These fish are unique among cichlids in the way they care for their young. Both males and females produce mucus on their body surfaces, which is the first food absolutely necessary for larval survival and growth [22,23,24,25]. The red discus grows up to 20 cm. Its characteristic feature is a strong lateral flattening of the body into an oval shape [14]. In wild individuals, the sides of the body are flesh pink, with numerous horizontal carmine lines. On the sides of the body, there are vertical stripes of varying intensity. The most prominent are three. The first is on the head (starting at the top of the head, passing through the fish’s eye, and ending under the gill lids), the second is in the middle of the body and runs from the center of the dorsal fin to the anal fin; and the third is on the shaft of the caudal fin. Characteristic of this species is the structure of the ventral fins—thin with a strongly elongated first ray. The ventral, dorsal, and anal fins are orange–red–blue. Males are almost indistinguishable from females. During spawning, the males develop short and sharply pointed decapods, while the decapods of the females are longer and tubular.

The studied cichlid species are harvested both for consumption purposes and for use in aquariums. Since the jaguar cichlid and green terror reach significant sizes, local fishermen eagerly catch and sell them in local markets for consumption purposes [26]. The green terror has tasty and delicate meat [27]. It is also a species farmed for consumption. A meat analysis showed significant differences in the quality of meat between fillets from farmed and wild fish [27]. The red discus is not a significant fish from a fisheries perspective, although anthropologists note its minor role in the diets of local communities.

However, the red discus is extensively caught for aquarium purposes because the red discus is among the most popular and frequently purchased ornamental fish in Europe and North America. Wild fish are highly valued by breeders due to their unique coloring which is hard to achieve under farm conditions, which makes wild fish reach prices several times higher (up to USD 500 per piece) than farm-raised fish. The jaguar cichlid and the green terror are also harvested for aquarium purposes; however, due to the lower prices of wild individuals, they are not harvested on as large a scale as the red discus.

Fishing pressure for both consumption and aquarium purposes, combined with habitat destruction and increasing water pollution, has led to a drastic reduction in the natural populations of the studied species [26,28,29]. The lack of countermeasures against the causes of the decline in these species could even lead to their extinction [30,31].

From the available publications addressing the reproduction of the studied cichlid species, we learn that Resende [32] estimated the sexual maturity and growth rate of jaguar cichlids from the wild (from rivers flowing through Brazil). They examined 520 individuals with an average length of 13.6 cm. Of the group of individuals studied, 72.64% of the fish were adults. It was found that the youngest mature and spawning individuals were on average 0.5 years old. The study of the speed and rate of gonad development in female jaguar cichlids showed that the reproductive strategy of jaguar cichlids is based on asynchronous ovarian maturation, with mature oocytes being located in the ovary next to the next batch of rapidly maturing oocytes. This allows these cichlids to maximize the time between spawning and, as a result, efficiently colonize successive reaches of the Grijalva–Usumacinta River [1]. Prazdnikov [33] studied the effects of triiodothyronine (T3) on the development of sexual characteristics, fecundity, and spawning behavior, as well as on embryonic development (hatching time, survival, and malformations) of the green terror. The relationship between the effects of T3 and a decrease in the number of eggs laid and an increase in the length of time between each spawning was demonstrated. The compound was also shown to have no effect on hatching time, larval survival, or incidence of developmental defects. Detailed studies on the development of the gonads of male and female *Andinoacara rivulatus* and an analysis of the hormones in the blood have shown that temperature, amount of light, and precipitation play a fundamental role in the processes of oogenesis and spermatogenesis in fish, and these relationships can be used to control the reproduction of this species for conservation [34]. The reproductive and embryonic development of *Symphysodon discus* is still not understood. Fragmentary knowledge of the biology of this species can be found only in popular scientific studies, but the embryonic and larval development of these fish has not been described. Despite their increasing popularity among aquarists, the reproduction of cichlids native to South and Central America has not been comprehensively described. Therefore, in this work, we focused our attention on learning, documenting, and describing in detail the reproductive strategy, the course of embryogenesis, and the development of larvae of the three studied cichlid species, selected for their similar reproductive strategy and for inhabiting similar aquatic environments (coastal zones of rivers). The results may be very useful for the development of reproductive biotechnology of the studied cichlid fish species, which can be used to the protection of these fish in theier natural habitat as well as in the world aquaculture. This could help satisfy the demand for these fish for fishkeeping and contribute to the conservation of these species in their natural environment.

## 2. Materials

### 2.1. Collection of Spawners

Adult fish of three species of cichlids (*Cichlidae*), namely the jaguar cichlid (*Parachromis managuensis*), the green terror (*Andinoacara rivulatus*), and the red discus (*Symphysodon discus*) were imported from the wild by a wholesaler (Akwen). The fish were transported and released into three spawning aquariums (each species separately) located in the aquarium rooms of the Faculty of Food Science and Fisheries at the West Pomeranian University of Technology in Szczecin with 10 fish of each species.

### 2.2. Acclimatization and Preparation of Fish for Spawning

The spawners were acclimatized in spawning aquariums, where the conditions were as close as possible to the natural environment, and the spawning pairs were allowed to mate. The fish were fed with dried spirulina (Tropical, Przemyśl, Poland), brine shrimp (*Artemia salina*), *Mysidacea* shrimp-like crustaceans, and northern shrimp (*Pandalus borealis*), which provided a varied diet so that the fish were in good condition and eager to spawn.

Three aquariums with dimensions of 150 × 50 × 60 cm and a capacity of 450 L were aerated by a compressor (Yasunaga Thomas P-60, Tokyo, Japan), and an adequate water quality was ensured by a filter (Fluval FX6, Hagen, Germany) with a flow rate of 2300 L/h. The aquariums were lit with 17 W LED bulbs for 12 h per day (from 8 a.m. to 8 p.m.). The water in the aquariums was changed once a week at a rate of 30% of the total tank volume. A constant water temperature of 26 ± 0.2 °C was maintained in the tanks using 200 W heaters (Aquael, Dubowo Drugie, Poland). In each of the three aquaria, the spawning pairs were prepared with five sturdy ceramic spawning cones (Tropical, Przemyśl, Poland) 25 cm tall, with a base diameter of 13 cm, and a very rough surface, which served as a hard substrate on which the fish spawned. The fish tanks were located in a separate room, isolated from external factors that could cause stress to the fish.

### 2.3. Experimental Procedure

Developing eggs from five pairs of adult cichlids of each species were studied. The developing eggs came from the fourth subsequent spawning. The purpose of this procedure was to eliminate the possibility of eggs of different sizes in the first spawning and to improve the survival rate of the eggs, since the spawning pair had already gained experience in caring for the offspring.

When spawning behavior was observed, which was manifested by intensive cleaning of the spawning cone by the parent pair, it was regularly checked every 30 min to see if the fish had started spawning. After spawning, the number of spawned eggs was photographed and counted. Then, 300 fertilized eggs were collected with a 2 ml pipette, and the test material was transferred to a 500 ml container with water from the aquarium where the spawning pairs were located. The 500 ml container was aerated with an aeration cube to ensure continuous movement of the water and sufficient oxygenation.

## 3. Methods Used

### 3.1. Analysis of Eggshell Structure

Eggs found immediately after activation in water were fixed in a 4% formalin solution. The fixed samples were dehydrated in an increasing series of alcohols and then in acetone. The eggs thus prepared were dried with liquid CO_2_ at the so-called critical point, after which the samples were mounted on saucers and sputtered with a thin layer of gold and palladium alloy (Polaron SC7620 Mini Sputter Coater, Laughton, UK). The slides were then observed using a scanning microscope (SU8000, Hitachi, Japan), and images of the egg cases were stored in a computer’s memory for later analysis.

### 3.2. Observation of Developing Embryos

The developing eggs were observed and photographed using a light microscope (Nikon 2000SE, Tokyo, Japan) and a stereoscopic microscope (Carl Zeiss Stereo Discovery.V12, Jena, Germany) with software to record images into computer memory from fertilization through larval hatching to complete yolk-sac resorption. Three eggs at a time were randomly selected from a container and placed in a Petri dish. The frequency of observation of the eggs varied according to the ontogenetic stage. After fertilization, the eggs were observed and photographed every hour for the first 20 h (until the onset of gastrulation), and every 5 h until the larvae hatched. In contrast, hatched larvae were observed every 12 h until complete resorption of the yolk sac and transition from endogenous to exogenous feeding. Eggs and larvae, after being photographed, were placed in separate containers and were not considered in further observations, as potential short-term manipulation could lead to developmental disturbances. No increase in the mortality of the eggs and larvae was observed after the manipulations. The observation time of the eggs and larvae was recorded both in hours post-fertilization (Hpf) and converted to degree hours (°H). A degree hour (°H) is the number of hours of embryogenesis multiplied by the average water temperature in which the fertilized eggs are incubating.

Additionally, during the course of organogenesis, the heart rate was observed and recorded every 2 h for 4 randomly selected developing embryos from the moment the heart appeared until the end of embryogenesis for a duration of 3 min each. During the observation, the number of heart contractions per minute was counted, and the results were averaged.

Yolk-sphere and egg diameters and eggshell thicknesses were measured using the specialized MultiScan program. Egg and yolk-sphere volumes were calculated using the formula (V = 1/6 × Π × d_1_ × d_2_^2^). The measurement results were compared using the Fisher’s test and Tukey’s test. For larvae, total body length (*longitudo totalis*—l.t.), yolk-sac length (l), and height (h) were measured. Measurements of diameters were taken using 20 eggs from each spawn (a total of 100 eggs for each species).

## 4. Results

### 4.1. The Spawning Process

Spawning of the studied species (jaguar cichlid, green terror, and red discus) began with intensive cleaning of the surface of the spawning cone. Observations of fish before spawning showed that the fish intensively cleaned the spawning site for 4 to 12 h, where they later deposited the eggs. The length of the cleaning period depended on the amount of eggs laid by the female. This is related to the cleaning of a larger surface area of the spawning cone on which the eggs were deposited. No spectacular pre-spawn mating dance was observed by the parental pair.

The spawning process was similar in each of the three species studied. Eggs were deposited in batches using an extended decapod, in vertical columns of dozens of grains per column. The columns were always confined to a single area within the pre-cleaned area where the parental pair spawned. These areas varied in size depending on the size of the female. After the female laid a column of eggs, the male immediately proceeded to pour milt over the eggs, starting at the bottom of the column. The male was not found to be pouring sperm on the eggs at the time the females were laying them.

The number of eggs laid depended on the species; the highest number of eggs per female was observed in jaguar cichlids, ranging from 2437 to 3427 eggs (average of 2991 eggs). Green terror spawners laid from 545 to 1230 eggs (average of 922 eggs), and the lowest number of eggs laid was observed in the red discus, from 250 to 350 eggs (average of 300 eggs).

### 4.2. Characteristics of Eggs

**Figure 1:**
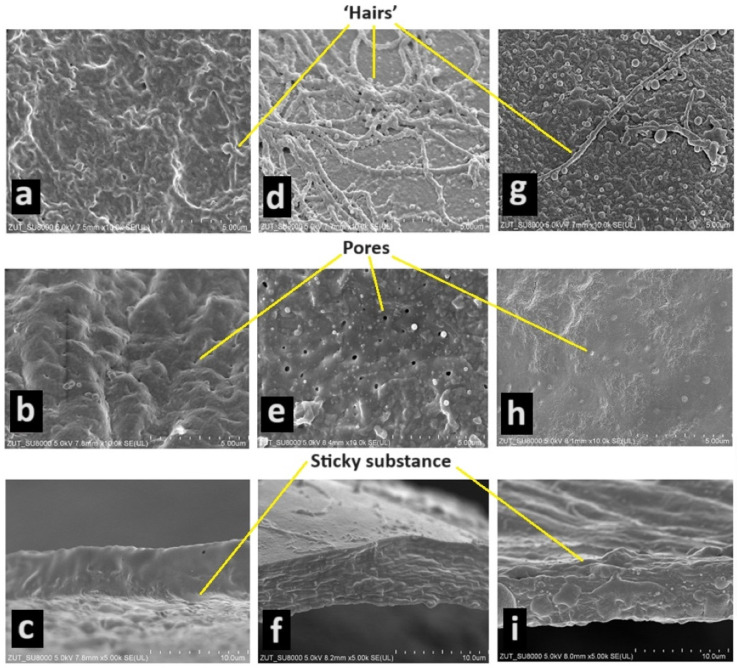
The eggshell of the jaguar cichlid (*Parachromis managuensis*) ((**a**)—view of outer surface of shell—magnification ×10,000, (**b**)—view of inner surface of shell—×10,000, (**c**)—cross-section through shell—×5000); the green terror (*Andinoacara rivulatus*) ((**d**)—view of outer surface of shell—×10,000, (**e**)—view of inner surface of shell—×10,000, (**f**)—cross-section through shell—×5000); the red discus (*Symphysodon discus*) ((**g**)—view of outer surface of shell—×10,000, (**h**)—view of inner surface of shell—×10,000, (**i**)—cross-section through shell—×5000).

The eggshells of the three species studied are sticky only during the first few seconds after the female lays her eggs. Detached eggs show no tendency to stick to the substrate or each other. The most sticky substance was found in the jaguar cichlid (Figure 1a,c). On the surface of the eggshell of the green terror, one can see a very large number of ‘hairs’ (Figure 1d), in the red discus a little less (Figure 1g), and in the jaguar cichlid they were not observed at all (Figure 1a). Thanks to this, in addition to the sticky substance, the eggs are attached to the hard substrate. On the inside of the eggshell, numerous irregularly distributed pores were observed. In the jaguar cichlid, there were the most pores on average per 1 um^2^—3.6 pores (Figure 1a); in the green terror, there were slightly fewer pores, on average 2.8 pores per 1 um^2^ (Figure 1e); and in the red discuss, there were the fewest pores, on average 1.6 pores per 1 um^2^ (Figure 1h). The thickness of the eggshell was also measured in the species studied. The green terror and jaguar cichlids had an identical eggshell thickness (0.0065 mm) (Figure 1c,f). In contrast, the red discus had a much thinner shell (0.0043 mm) (Figure 1i).

**Figure 2:**
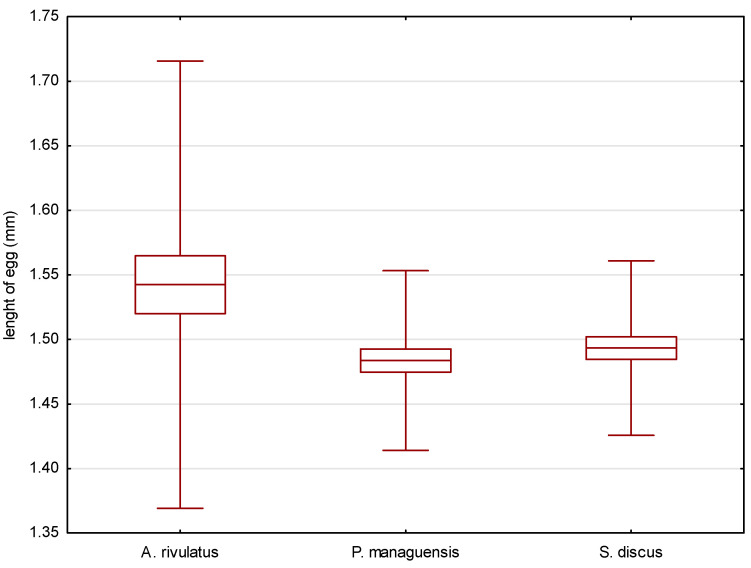
Comparison of the average length of the eggs of the studied species. *p* = 0.01548.

**Figure 3:**
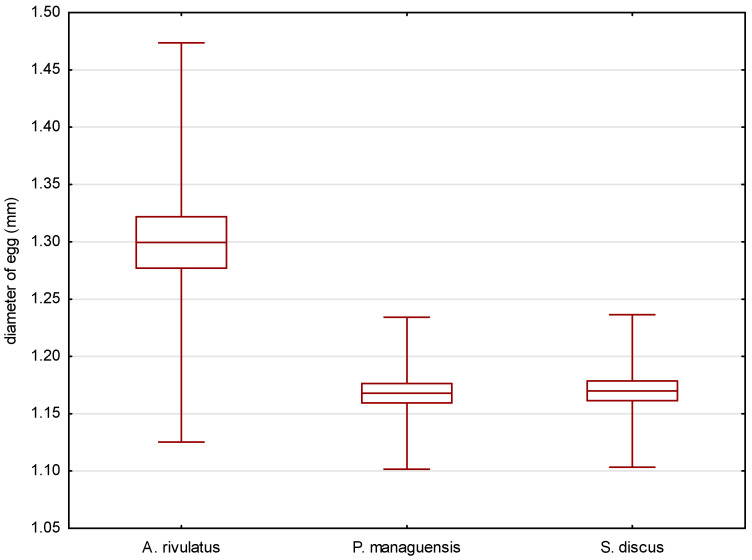
Comparison of the average width of the eggs of the studied species. *p* < 0.00001.

**Figure 4:**
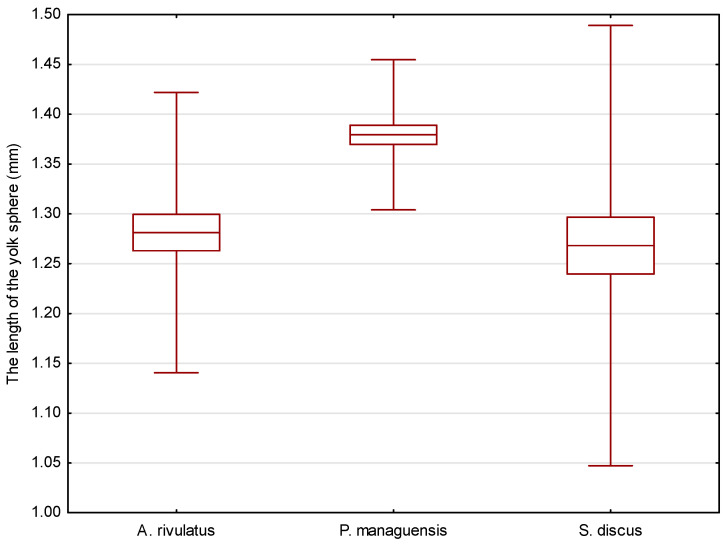
Comparison of the average yolk sphere length of the studied species. *p* = 0.00058.

**Figure 5:**
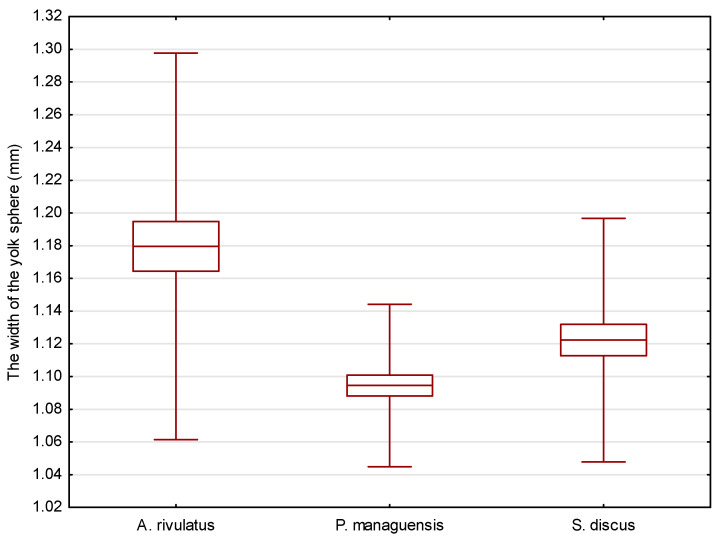
Comparison of the average yolk sphere width of the studied species. *p* = 0.00001.

The eggs of the jaguar cichlid were transparent with a beige color. They were oval in shape, with diameters of 1.484 ± 0.035 mm (Figure 2) and 1.168 ± 0.033 mm (Figure 3). The egg volume was 1.060 ± 0.050 mm^3^. The oval-shaped yolk sphere, with diameters of 1.379 ± 0.038 mm (Figure 4) and 1.095 ± 0.025 mm (Figure 5), was 0.865 ± 0.43 mm^3^ when the volume was calculated. Both the yolk sphere and the eggs varied in size, as the difference between the smallest and largest eggs was 75%. The average perivitellar space was 18%. Numerous irregularly distributed adipocytes were observed around the yolk sphere.

The eggs of the green terror were transparent with a yellow–beige color. The oval-shaped eggs had diameters of 1.542 ± 0.087 mm (Figure 2) and 1.299 ± 0.087 mm (Figure 3), respectively. The egg volume was 1.365 ± 0.16 mm^3^. The oval-shaped yolk sphere, with diameters of 1.281 ± 0.070 mm (Figure 4) and 1.180 ± 0.059 mm (Figure 5), was 0.935 ± 0.10 mm^3^ when the volume was calculated. Both the yolk sphere and the eggs were differentiated, as the difference between the smallest and largest eggs was 61%. The average perivitellar space was 31%. Numerous irregularly distributed fat droplets were observed on the yolk sphere.

The amber-colored eggs of the red discus were oval in shape, with diameters averaging 1.493 ± 0.034 mm (Figure 2) and 1.170 ± 0.033 mm (Figure 3), which when converted to volume was 1.070 ± 0.054 mm^3^. The oval-shaped yolk sphere had a diameter of 1.268 ± 0.110 mm (Figure 4) and 1.122 ± 0.037 mm (Figure 5). The volume was 0.836 ± 0.088 mm^3^. Both the yolk sphere and the eggs were differentiated, as the difference between the smallest and largest eggs was 70%. The perivitellar space averaged 22%. Numerous irregularly scattered fat droplets were observed on the yolk sphere.

The conducted statistical tests revealed significant differences in the length of the measured egg diameters between the green terror and the jaguar cichlid, as well as between the green terror and the red discus. However, no statistically significant differences were found between the jaguar cichlid and the red discus. By analyzing the size of the yolk sphere, a statistical difference was found between the jaguar cichlid and the red discus, as well as between the jaguar cichlid and the green terror. However, no statistically significant differences were found between the green terror and the red discus in terms of the longer diameter. For the shorter diameter of the yolk sphere, statistical differences were found between the green terror and the jaguar cichlid, as well as between the green terror and the red discus, but no statistically significant differences were found between the jaguar cichlid and the red discus.

### 4.3. Embryonic Development

To describe embryogenesis, three periods of embryonic development were selected and divided into furrowing, gastrulation, and organogenesis. Then, the hatching of the studied cichlid fish species was characterized.

### 4.4. Cleavage

**Figure 6:**
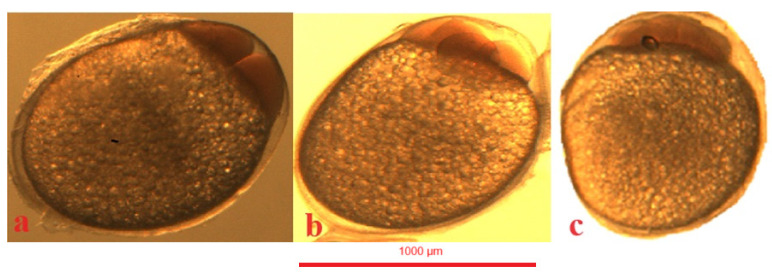
Jaguar cichlid (*Parachromis managuensis*): (**a**)—stage of two blastomeres, (**b**)—stage of four blastomeres, (**c**)—stage of eight blastomeres.

In the jaguar cichlid, 2 blastomeres were observed at 43 °H (Figure 6a), 4 blastomeres at 56 °H (Figure 6b), 8 blastomeres at 69 °H (Figure 6c), and 16 blastomeres at 82 °H. The blastula stage was observed at 520 °H (Table 1).

**Figure 7:**
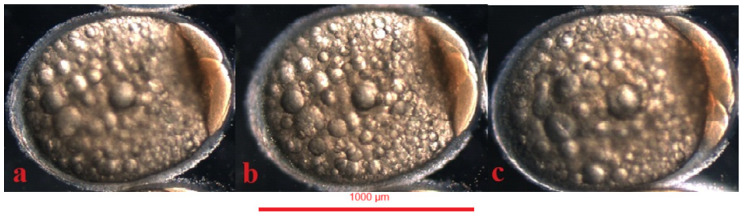
Green terror (*Andinoacara rivulatus*): (**a**)—stage of two blastomeres, (**b**)—stage of four blastomeres, (**c**)—stage of eight blastomeres.

In the green terror, 2 blastomeres were observed at 39 °H (Figure 7a), 4 blastomeres at 52 °H (Figure 7b), eight blastomeres at 61 °H (Figure 7c), and 16 blastomeres at 78 °H. The blastula stage was observed at 468 °H (Table 1).

**Figure 8:**
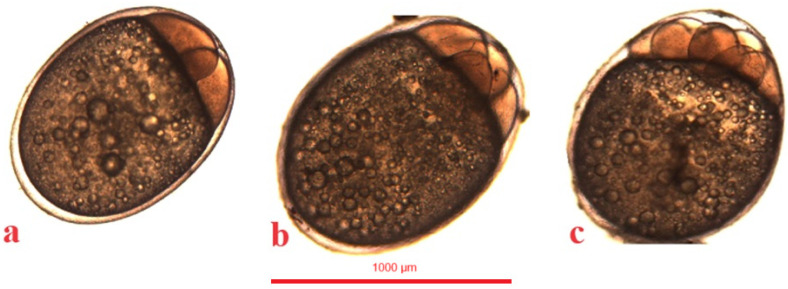
Red discus (*Symphysodon discus*): (**a**)—stage of two blastomeres, (**b**)—stage of four blastomeres, (**c**)—stage of eight blastomeres.

In the red discus, 2 blastomeres were observed at 26 °H (Figure 8a), 4 blastomeres at 39 °H (Figure 8b), eight blastomeres at 53 °H (Figure 8c), and 16 blastomeres were observed at 65 °H. The blastula stage was observed at 455 °H (Table 1).

### 4.5. Gastrulation

**Figure 9:**
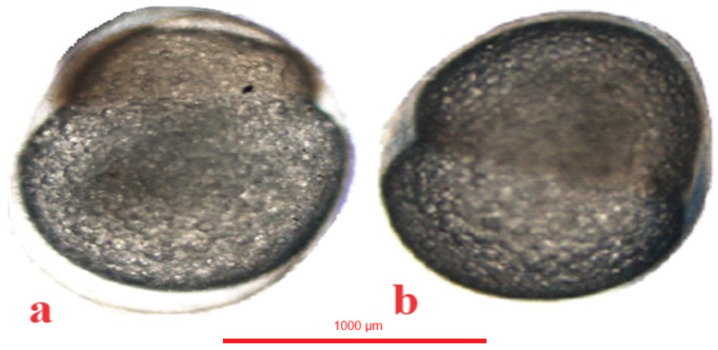
Jaguar cichlid (*Parachromis managuensis*): (**a**)—epiboly ⅓, (**b**)—epiboly ¾.

In the jaguar cichlid, the gastrulation period began at 520 °H, that is, 20 h after fertilization. At 598 °H a one-third epiboly was visible (Figure 9a), at 728 °H a one-half epiboly, and at 910 °H a three-fourths epiboly (Figure 9b). Gastrulation ended at 1066 °H, with the yolk globule undergoing a complete epiboly (Table 1).

**Figure 10:**
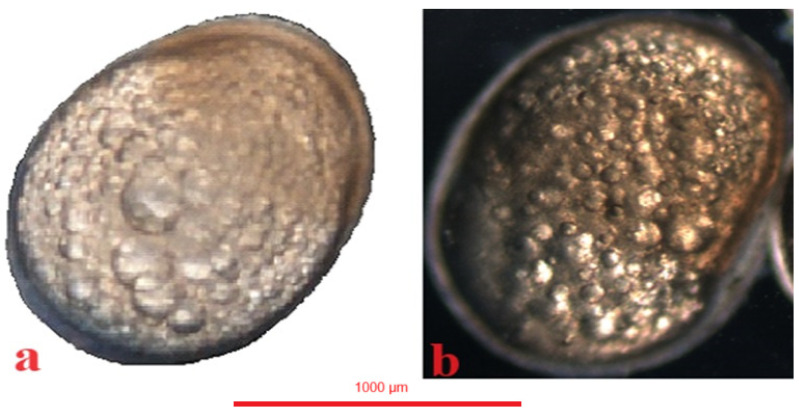
Green terror (*Andinoacara rivulatus*): (**a**)—epiboly ⅓, (**b**)—epiboly ¾.

In the green terror, the gastrulation period began at 468 °H, that is, 18 h after fertilization. At 572 °H, a one-third epiboly was visible (Figure 10a), at 650 °H a one-half epiboly was visible, and at 741 °H a three-fourths epiboly was visible (Figure 10b). Gastrulation in the green terror ended at 858 °H, with the completed epiboly of the yolk sphere (Table 1).

**Figure 11:**
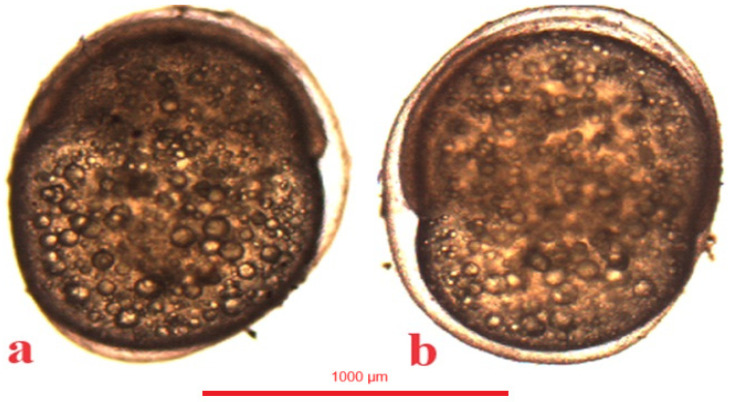
Red discus (*Symphysodon discus*): (**a**)—epiboly ⅓, (**b**)—epiboly ½.

In the red discus, the gastrulation period begins at 455 °H, which is 17.5 h after fertilization. An epiboly one-third (Figure 11a) is visible at 546 °H. At 637 °H, a half-grown yolk was observed, and at 728 °H a three-fourths epiboly is visible (Figure 11b). Gastrulation in the red discus ends at 845 °H with complete epiboly of the yolk sphere (Table 1).

### 4.6. Organogenesis

**Figure 12:**
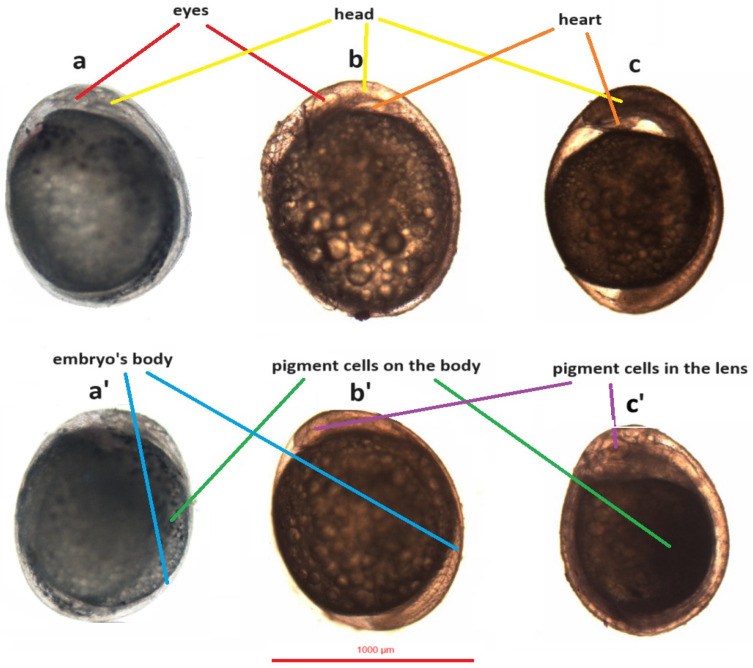
Organogenesis in the studied species. (**a**,**a’**)—jaguar cichlid (*Parachromis managuensis*), (**b**,**b’**)—green terror (*Andinoacara rivulatus*), (**c**,**c’**)—red discus (*Symphysodon discus*).

In the jaguar cichlid, the cephalic part and the dorsal string became visible at 1118 °H. Then, after an average of 5 h at 1248 °H, the eyeball ovary became visible. At 55.5 (1443 °H) hours after fertilization, slow and irregular heart contractions (average 36 beats per minute) were observed. After another 8 h, the heart contractions became more regular and faster (average 60 beats per minute), and at 1651 °H the first movement of the embryo was observed. At 64.5 h (1677 °H) after fertilization, a braincase was observed in the cephalic part of the jaguar cichlid. At 66 h (1716 °H) post-fertilization, pigment became visible in the lens, and after another 3 h, pigment was observed on the sides of the yolk sac (Table 1, Figure 12a,a’).

In the green terror, the cephalic part and the dorsal string become visible at 1040 °H. Then, after an average of 3 h at 1118 °H, the eyeball ovary becomes visible. At 45 (1170 °H) hours after fertilization, slow and irregular heart contractions (55 beats per minute) were observed. After another 9 h, the heart contractions became more regular and faster (average 80 beats per minute), and at 1404 °H, the first movement of the embryo was observed. At 56 h after fertilization (1456 °H), a braincase was observed in the cephalic part of the green terror. At 58 h post-fertilization (1508 °H), the pigment was visible in the lens, and after another 16 h, the pigment was observed on the sides of the yolk sac (Table 1, Figure 12b,b’).

In the red discus, the cephalic part and the dorsal string become visible at 1014 °H. Then, after an average of 3 h at 1092 °H, the eyeball ovary becomes visible. At 42.5 (1105 °H) hours after fertilization, slow and irregular heart contractions (45 beats per minute) were observed. After another 9 h, the heart contractions became more regular and faster (65 beats per minute), and at 1339 °H, the first movement of the embryo was observed. At 55.5 h after fertilization (1443 °H), a braincase was observed in the cephalic part of the red discus. At 57.5 (1495 °H) hours after fertilization, pigment became visible in the lens, and after another 14.5 h, pigment was observed on the sides of the yolk sac (Table 1, Figure 12c,c’).

### 4.7. Hatching

**Figure 13:**
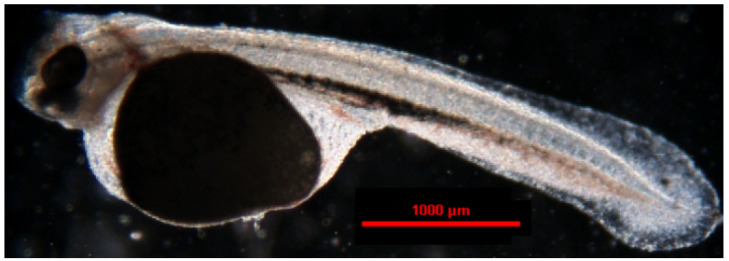
The larva of the jaguar cichlid (*Parachromis managuensis*).

In the jaguar cichlid, the first hatched larvae (Figure 13) were observed 83 h after spawning (2158 °H). After another 3 h, 50% of the embryos had hatched. All larvae that emerged from the egg cases were observed 90 h after fertilization (2340 °H) (Table 2). Jaguar cichlid larvae measured an average length of 5.301 ± 0.22 mm. After hatching, the larvae lay freely on the bottom of the container with intense body movements, but they were unable to actively swim. The yolk sac measured an average of 1.998 ± 0.31 mm in length and 1.345 ± 0.04 mm in width. The first attempts to swim and feed were observed 5 days after the larvae hatched. The fish were fed with freshly hatched artemia. The yolk sac was completely resorbed after 6 days. After this time, the fish began to swim and feed freely (Table 3).

**Figure 14:**
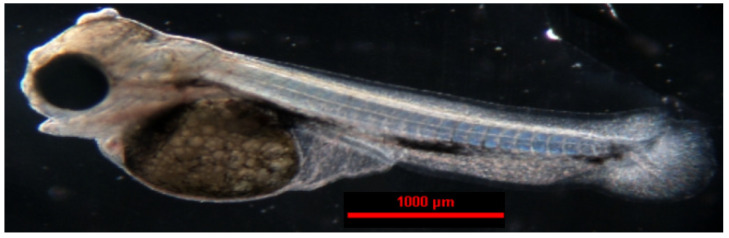
The larva of the green terror (*Andinoacara rivulatus*).

In the green terror, the first individual larvae (Figure 14) were observed 87 h after spawning (2262 °H). After another 4 h, 50% of the embryos had hatched. All larvae that emerged from the eggshells were observed 95 h after fertilization (2470 °H) (Table 2). The larvae had an average length of 5.203 ± 0.022 mm. The hatched larvae lay on the bottoms of the boxes with intense body movements, but they did not swim. The yolk sac measured 1.554 ± 0.083 mm in length and 1.182 ± 0.007 mm in width. On the sixth day after hatching, swimming and feeding attempts were observed. The larvae completely resorbed the yolk sac after 7 days and foraged intensively (Table 4).

**Figure 15:**
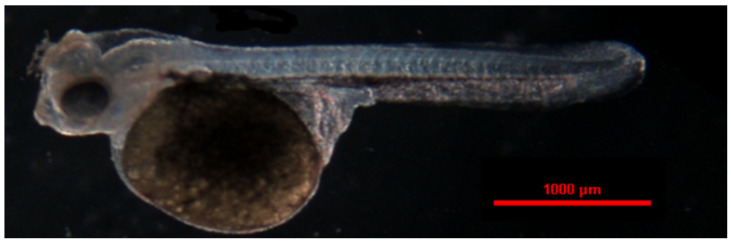
The larva of the red discus (*Symphysodon discus*).

In the red discus, the first individual larvae were observed 82 h after spawning (2132 °H) (Figure 15). After another 3 h, 50% of the embryos hatched. All larvae left the eggshell at 89 h after fertilization (2314 °H) (Table 2). The mean length of the larvae was 4.346 ± 0.027 mm. The hatched larvae lay on the bottom of the container with intense body movements, but they did not swim. The large yolk sac averaged 1.403 ± 0.031 mm in length and 1.112 ± 0.011 mm in width. The fish began to attempt to swim and feed 3 days after hatching. The larvae completely resorbed the yolk sac one day later (4 days after hatching) and foraged intensively (Table 5).

## 5. Discussion

The spawning behaviors observed during the experiments are characteristic of fish that lay eggs on hard substrates, e.g., in *Cichlasoma nigrofasciatum*, *Coptodon zillii*, *Laetacara araguaiae*, *Amphilophus* sp., and *Amphiprion ocellaris* [35,36,37,38,39].

The duration of spawning in the observed species depended on the size of the fish and the number of eggs laid. The larger the parental individuals were, the longer the spawning lasted, and the more eggs were laid. This phenomenon has also been observed in other lithophilic fishes such as *Salmo salar* or *Salmo trutta* [40,41]. The significant differences observed in the average number of eggs laid by the female were due to the size and shape of the fish body and most likely related to the size of the ovaries [1,34]. A relationship between body size and number of mature eggs has also been observed in other cichlids (*Cichlasoma nigrofasciatum*, *Coptodon zillii*, and *Cichlasoma orientale*) [35,36,42,43], while in *Cichlasoma urophthalmus*, no correlation between female size and number of mature eggs has been demonstrated [44].

A highly developed parental instinct can be observed in the studied species. After spawning, the spawners take care of the deposited eggs by fanning them intensely with their pectoral fins, causing an intense circulation of oxygen-rich water, which is necessary for proper embryonic development [37,45].

The color of the eggs (amber) observed in the red discus and green terror cichlid is the most commonly encountered hue among fish of the cichlid family. It has been previously described in other cichlids such as *Symphysodon aequifasciatus* [29] and *Astronotus ocellatus* [46]. Less frequently, it is olive colored, as in *Coptodon zillii* [47]. Eggs of the jaguar cichlid are characterized by a distinctly rarer beige color. Significant differences in the sizes of the eggs among the studied species are also characteristic within the cichlid family. This phenomenon has been observed in *Haplochromis xenognathus*, *H. nyererei*, *H. pyrrhocephalus*, *H. velvet black*, and *H. theliodon* [48]. One of the species with the largest eggs is *Astronotus ocellatus* [46].

Two blastomeres and the subsequent stages of embryogenesis were observed the earliest in the red discus, which may be related to the biology of this fish, which often lives in very warm waters, resulting in biological memory in this species [19]. The rapid onset of blastomere divisions after fertilization has also been observed in the closely related species *Symphysodon aequifasciatus* [29]. In the studied fish species, distinct differences in heart rate were observed at the same stage of organogenesis. At the initial stage, the heart worked slowly and irregularly, but as the embryo developed, the heart rate increased, and its contractions became regular. A similar phenomenon was observed in another cichlid fish (*Pterophyllum scalare*) [49]. In the jaguar cichlid, the slowest heart rate was observed, which could be due to the slower progression of embryogenesis. However, it is intriguing that, in the green terror cichlid, the heart rate was faster than in the red discus, despite the slower embryogenesis in this species compared to the red discus. The first species in which pigmented cells were found in the eyes was the red discus, followed by the observation of pigmented cells in the eyes of the green terror, and finally in the jaguar cichlid. This stage is called oviposition and is very important for safe egg transfer. From the time the eggs are oviposited until they hatch, the loss of eggs is very small [50,51]. Also during this period, dead eggs can be observed, which are mainly characterized by their white color [52]. Pigment cells on the yolk sac and dorsal part were observed earliest in the jaguar cichlid, followed by the red discus and the green terror. This is due to the natural coloration of these fish, as adult jaguar cichlids are dark, even black, during spawning [9].

Regarding the duration of hatching in all three species, a large time span was observed in the hatching of larvae. Similarly long hatching has been observed in *Amphiprion ocellaris* [39], where larvae took several hours to hatch, probably due to the prolonged spawning of the fish and egg laying by adults in portions.

Significant differences in larval size were also found. The species with the smallest larvae were larvae of the red discus, which is related to the shortest course of embryogenesis. The largest larvae were found in the jaguar cichlid, which had the longest embryogenesis. The duration of embryogenesis affects the size of cichlid larvae, as the larvae can incubate longer in the egg and thus draw more nutrients from the yolk sac. The red discus had the smallest yolk-sac volume, while the jaguar cichlid had the largest yolk-sac volume. Researchers came to similar conclusions by conducting experiments on other fish and animals. They proved that the length of embryonic development affects larval size and survival in both freshwater and marine fish [48,53,54,55].

## 6. Conclusions

The article provides a detailed description of the knowledge and characterization of the reproductive strategy, embryonic development, and larval development in the jaguar cichlid (*Parachromis managuensis*), the green terror (*Andinoacara rivulatus*), and the red discus fish (*Symphysodon discus*). The most significant conclusions that can be drawn are as follows. The duration of embryogenesis and yolk-sac resorption in the studied species, which allows the breeder to determine the hatching time and the time when the fish begin to feed. The number of eggs laid during spawning by the studied species has also been determined, which will contribute to better breeding planning, production volume, and fish rearing by breeders. Another important conclusion is the determination of the moment of eyeing the eggs, which allows the breeder to safely transfer the incubating eggs. Additionally, thanks to a comprehensive analysis of the embryogenesis, each breeder and scientist can draw their own conclusions depending on their individual needs. This manuscript has contributed to the development of biotechniques for breeding the jaguar cichlid (*Parachromis managuensis*), the green terror (*Andinoacara rivulatus*), and the red discus fish *(Symphysodon discus*). This will enable effective and easy breeding of these fish under controlled conditions, which will not only satisfy the demand for these species in the aquarium trade but also protect them in their natural environment by discontinuing fishing activities. Further research on the embryogenesis of the aforementioned species is currently planned, taking into account the influence of physicochemical factors, such as temperature, salinity, the impact of commonly used antiseptics, and the influence of magnetic fields, on developing eggs.

## Figures and Tables

**Table 1:** Time course of embryogenesis in jaguar cichlid (*Parachromis managuensis*), green terror (*Andinoacara rivulatus*), and red discus (*Symphysodon discus*).

		Jaguar Cichlid		Green Terror		Red Discus	
		Degree Hour (°H)	Hour (Hpf)	Degree Hour (°H)	Hour (Hpf)	Degree Hour (°H)	Hour (Hpf)
Fertilization		0	0	0	0	0	0
Cleavage	2 blastomeres	43	1.66	39	1.5	26	1
	4 blastomeres	56	2.16	52	2	39	1.5
	8 blastomeres	69	2.66	61	2.35	52	2
	16 blastomeres	82	3.16	78	3	65	2.5
Gastrulation	beginning	520	20	468	18	455	17.5
	epiboly 1/3	598	23	572	22	546	21
	epiboly 2/3	728	28	650	25	637	24.5
	3/4 epiboly	910	35	741	28.5	728	28
	Closure of the blastopore	1066	41	858	33	845	32.5
Organogenesis	Making the head part visible	1118	43	1040	40	1014	39
	Eye primordia	1248	48	1118	43	1092	42
	First heart contractions	1443	55.5	1170	45	1105	42.5
	First movements of the embryo	1651	63.5	1404	54	1339	51.5
	Making the brain visible	1677	64.5	1456	56	1443	55.5
	Pigment cells become visible in the lens	1716	66	1508	58	1495	57.5
	The appearance of pigment cells on the body	1794	69	1924	74	1872	72

**Table 2:** Comparison of larval hatching time in jaguar cichlid (*Parachromis managuensis*), green terror (*Andinoacara rivulatus*), and red discus (*Symphysodon discus*).

		Red Discus		Jaguar Cichlid		Green Terror	
		Degree Hour (°H)	Hour (Hpf)	Degree Hour (°H)	Hour (Hpf)	Degree Hour(°H)	Hour (Hpf)
Hatching	First hatching	2132	82	2262	87	2158	83
	Hatching 50%	2210	85	2366	91	2236	86
	Hatching 100%	2314	89	2470	95	2340	90

**Table 3:** Length of larvae and yolk sac of jaguar cichlid (*Parachromis managuensis*) immediately after hatching and immediately after starting to swim.

Larvae after hatching			
Body length	Length of yolk sac	Width of the yolk sac	Volume of the yolk sac
5.301 mm	1.988 mm	1.345 mm	1.885 mm^3^
**Larvae after starting to swim**			
Body length	Length of yolk sac	Width of the yolk sac	Volume of the yolk sac
6.545 mm	1.518 mm	1.123 mm	1.024 mm^3^

**Table 4:** Length of larvae and yolk sac of green terror (*Andinoacara rivulatus*) after hatching and after starting to swim.

Larvae immediately after hatching			
Body length	Length of yolk sac	Width of the yolk sac	Volume of the yolk sac
5.203 mm	1.554 mm	1.182 mm	1.137 mm^3^
**Larvae immediately after starting to swim**			
Body length	Length of yolk sac	Width of the yolk sac	Volume of the yolk sac
6.136 mm	1.106 mm	0.754 mm	0.329 mm^3^

**Table 5:** Length of larvae and yolk sac of red discus (*Symphysodon discus*) after hatching and after starting to swim.

Larvae immediately after hatching			
Body length	Length of yolk sac	Width of the yolk sac	Volume of the yolk sac
4.346 mm	1.403 mm	1.112 mm	0.909 mm^3^
**Larvae immediately after starting to swim**			
Body length	Length of yolk sac	Width of the yolk sac	Volume of the yolk sac
5.447 mm	0.987 mm	0.882 mm	0.402 mm^3^

## Data Availability

The data generated and analyzed during the current study are available from the corresponding author upon reasonable request. The data are not publicly available due to the excessive amount of collected data.
